# Some Clinical Cases

**Published:** 1888-04

**Authors:** Geo. W. Sherbino

**Affiliations:** Abilene, Texas


### SOME CLINICAL CASES.

Geo. W. Sherbino, M. D., Abilene, Texas.

Dysmenorrhœa.—Miss —— has had painful menstruation for several years; is strong and healthy in other respects; is of of a lymphatic temperament. At the approach of the menses she becomes weary and languid; has headache and backache, more across the sacrum; aching all over the body; *deep aching in the bones; bed feels hard; becomes stupid and drowsy.* Tongue is coated yellow down the centre; no appetite; cannot digest her food; it makes her sick. Has "fever," sometimes delirious at night. Vertigo on rising up. (Acon., Bry., Phyt.) Feels better when keeping quiet (Bry.), *yet the bed gets so hard she must move to find a soft place.* Soreness in the uterine region and abdomen; bearing down. These symptoms all leave with the flow, to reappear at the next period.

Cured the case with Baptisia$^{cm.}$ in three months, giving a dose before the flow came on, followed by S. L. during the intervals.

Necrosis of the Lower Jaw.—Mrs. ——, a brunette, æt. forty, was taken with pain and swelling of the lower jaw in the region of the incisors; the swelling spreads backward, involving the whole jaw; it was so great as to evert the lower lip, and the whole face was swollen; there was also some fever present. The pain was not of a characteristic kind; the parts were red; the sleeplessness was very great, with starting and jumping in sleep. (Bell., Nux-v., Opium.) The case had gone on for several days without any improvement, when her friends, becoming dissatisfied, took the case from me and turned her over to the mercies of a "regular," who gave her Morphine and "Spiritus frumenti" *ad libitum.* She lost all her teeth in the lower jaw, from the first molar on one side to the first on the other, and some pieces of necrosed bone were extracted, leaving a large cavity, from which pus flowed profusely; her pulse was 132°, her temperature 100°; one can easily imagine her weak and wretched condition.

As the "regular" could do nothing for her relief, I was again called in; found her extremely weak and emaciated; as helpless as a child. In two weeks she was able to sit up; in three weeks she could ride out, and from this time she improved steadily. The first month she took three or four doses of Silicea$^{5m}$, with S. L.; then she had two doses of the CM (Fincke). The second month she received Hepar$^{12m}$, for the abuse of Mercury to which she had been subjected, and which caused excessive sensibility to touch, greatly aggravated on exposure to north or west winds. Hepar removed these symptoms and completed a permanent cure. Could surgery do more?

Typhoid.—Was called to see a married lady who had been sick for two weeks under old school treatment; as she grew steadily worse, her husband despaired of her recovery. The doctor told him she would never get well. I found the patient unconscious, muttering to herself, talking unintelligibly. She wanted to go home; thought she had been poisoned; imagined that she had been confined, and that a young baby was in the bed with her; that the doctor had poisoned it; was picking at the bed-clothes; had subsultus tendinum; there was marked tenderness in the right iliac region with a boggy feeling, bubbling against my finger on touch. She would give evidence of pain on pressure; bowels were constipated; had had a calomel purge a few days before, hence the constipation. The husband told me she had commenced her sickness with aching all over her body, and that she was so stupid the doctor could not arouse her; she would go to sleep while trying to answer him; she complained of pain in the back of her head down the spine; tongue had a heavy white coating; sordes on the teeth. I decided to give her one dose of Baptisia$^{cm}$ (Fincke), with S. L. every hour. Next day not quite so stupid, nor tongue quite so dry. Says some one shot her through the heart with a bullet. I have found this guiding symptom clinical—the patient squirts the water out of her mouth; this patient squirted the water clear across the bed. This made me think I was right in my remedy. Hering gives this symptom in his *Guiding Symptoms.*

She improved for several days on S. L.; the temperature came down from 103° to normal; one morning it was 96° in the axilla, 97° in the vagina. She had cold perspiration on her hands and arms; her pulse varied from 80° to 104°, never getting above 104°. Respiration was from thirty to forty per minute; the temperature would stay down a day or two and then go up again. It would go up and stay for a few days, then come down to sub-normal, 96°, with the cold hands and arms. Three times the temperature acted in this way, which is a very singular phenomenon, one I have never seen before; nor have I ever read of it. She was given the second dose of Baptisia as soon as the first had exhausted its action. This was all the medicine she had for two weeks. The temperature was very irregular. After the third week she knew every one who came to her bedside, especially in the morning. The symptoms seemed to change and the patient not to improve, but rather get worse again. There was the marked fan-like motion of the alæ nasi, which, indeed, had been present from the beginning. She was very restless, seemed worse from four to eight P. M.; no stools, and had used enema twice with no results. I had been using the catheter night and morning, as she had no power to urinate; the tongue was as dry as a chip; there was dropping of the lower jaw; gurgling in the left hypochondriac region; on saving some of the urine we found *red sand* in the bottom. I gave her one dose of Lycopodium$^{30m}$. For a week she did finely, jaw not so depressed nor the tongue so dry; urine not so scanty. One morning she defecated and urinated in the bed; the urine had passed in the bed before, about the time I commenced using the catheter. About this time the temperature again went up to 103°. So I gave another dose of Lyc.$^{30m}$; she had a fluctuating temperature for a few days more, when it came down to normal. The bowels acted for the first time* and the urinary organs were acting without assistance; the mind was clearing up, only when she had the cold spells, then she was flighty. I ought to have mentioned in the beginning that the diagnosis of the "regular" was ulceration of the womb. Before the patient was taken sick he had been dilating and cauterizing the cervix!! There was marked aggravation after the first dose of Lycopodium. Supposing I had repeated it every two hours, as is often the custom, I would probably have produced such a reaction that she could never have overcome it.

N. B.—This patient is a Bromidia eater; had been indulging heavily before she was taken sick. Some of her mental symptoms were probably due to this cause.

* NOTE.—The presence of constipation in cases of typhoid fever is to be regarded as a favorable symptom, and diarrhœa as the reverse. "I have not lost a single patient in whom the bowels remained costive up to the time of the crisis, when papescent stools afford relief."—JAHR.

"I said the same thing more than twenty years ago and can repeat it now. Hardly in the third week is the non-appearance of a stool to be regarded with any concern. The same applies to child-bed."—C. HERING.

